# An Unusual Presentation of Glioependymal Cyst Encroaching Neuronal Parenchyma in an Elderly Female: A Case Report

**DOI:** 10.7759/cureus.37835

**Published:** 2023-04-19

**Authors:** Amey M Bakshi, Aman Agrawal, Sanket S Bakshi, Anshool Kumbhare, Swarupa Chakole

**Affiliations:** 1 Department of Medicine, Jawaharlal Nehru Medical College, Datta Meghe Institute of Higher Education and Research, Wardha, IND; 2 Department of Community Medicine, Jawaharlal Nehru Medical College, Datta Meghe Institute of Higher Education and Research, Wardha, IND

**Keywords:** ventriculoperitoneal shunt, hydrocephalus, epithelial-lined, neuroepithelial cyst, glioependymal cyst

## Abstract

A glioependymal cyst (GEC) is a rare type of cyst that occurs within the brain and spinal cord. A 42-year-old male patient with a cystic lesion in the right frontal lobe was admitted to the hospital to have his headache, vertigo, and body spasms evaluated. MRI scans showed a mass in the right side of the frontal lobe which caused a mass effect over the lateral ventricle and corpus callosum. The patient became symptom-free after the craniotomy, followed by fenestration of cortices and cyst wall removal.

## Introduction

Glioependymal cysts (GECs) are a rare but significant type of cyst that can develop in the central nervous system. These cysts are characterized by both glial and ependymal cells, which are important types of cells that provide support and protection to nerve cells in the brain and spinal cord. While GECs are typically benign, they can cause a range of symptoms if they grow large enough to pressure surrounding tissues. These symptoms can include headache, seizure, and neurological deficits, significantly impacting a person’s life. Despite the importance of the cysts, relatively little is known about their underlying causes or the approach for diagnosis and treatment [1]. We present a rare case of a GEC that developed in the right frontal lobe, causing mass effects over other neuronal cells.

## Case presentation

**Figure 1:**
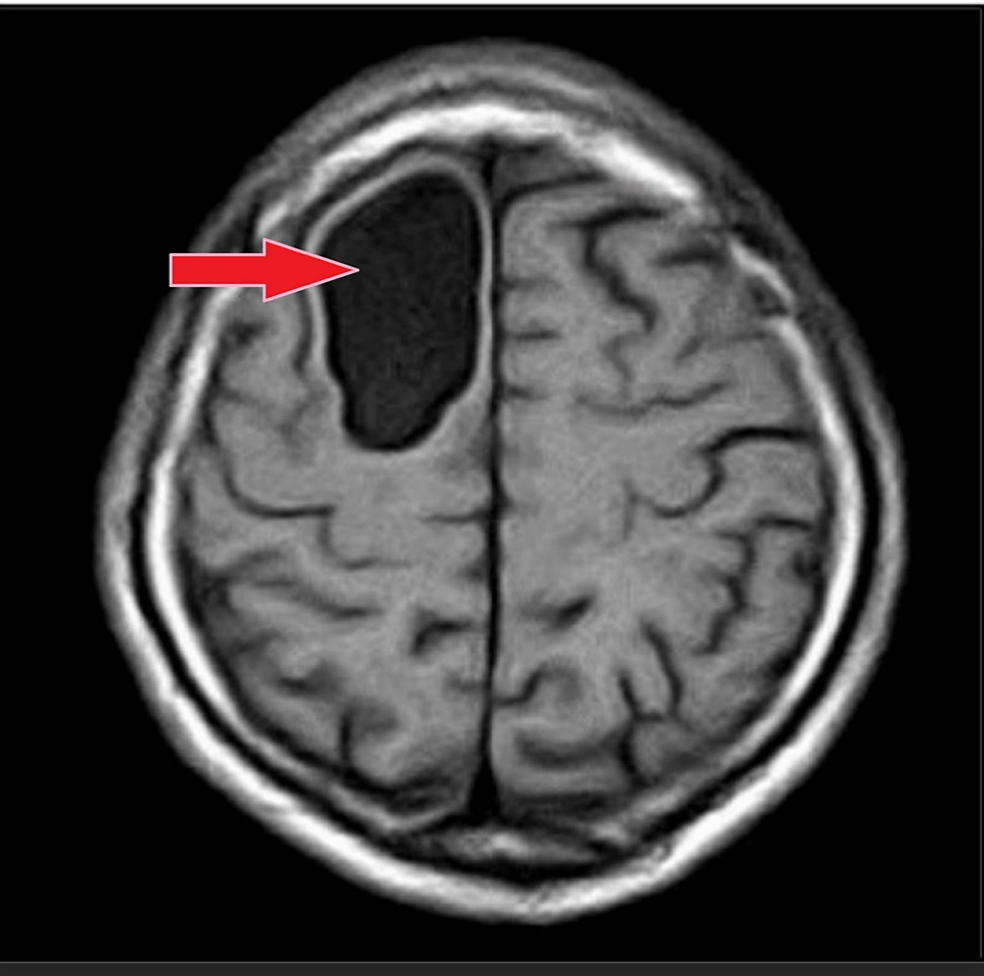
T1 axial section showing hypointense lesion

**Figure 2:**
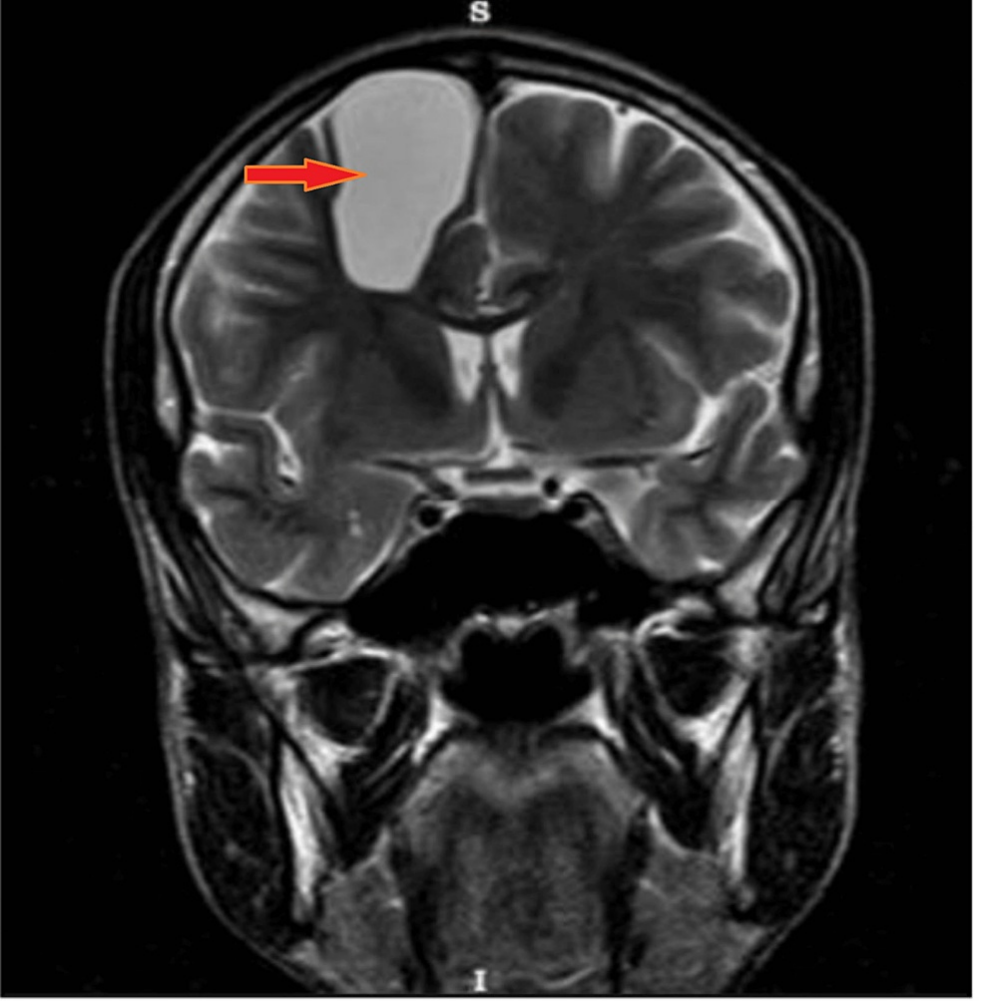
T2 coronal section showing hyperintense lesion

**Figure 3:**
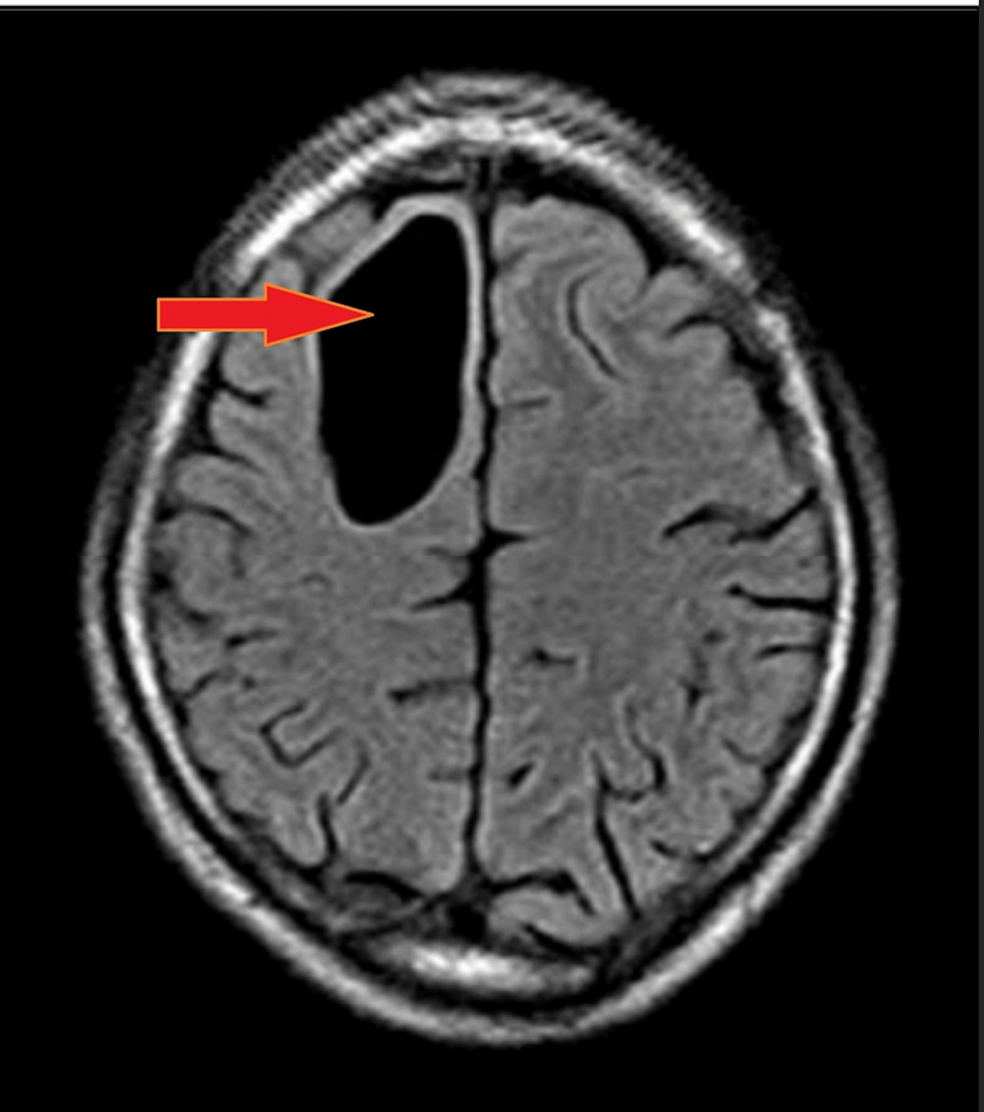
Flair axial section showing suppression of signal

This is a case of a 42-year-old female with a previous medical history of diabetes mellitus well controlled with antidiabetic medication. The patient presented to the emergency department with complaints of a sudden onset of intermittent headaches and dizziness for the past few months. He initially ignored the symptoms, attributing them to stress at work. However, the symptoms began to worsen and were accompanied by occasional episodes of nausea and vomiting. There was no history of unconsciousness, fever, or other medical conditions. The past history and family history of the patient remain insignificant. Physical and systemic examinations and all laboratory investigations were within normal limits. The patient’s EEG revealed a sharp wave pattern. On admission, an MRI was advised, which showed a 42 x 51 x 22 mm ( SI X TR X AP ) cyst. The margins were well-defined, and the lesion was oval on the right side of the frontal lobe with an intensity higher than that of CSF. This lesion was causing a mass effect over the surrounding neuroparenchyma and corpus callosum. No limited diffusion, calcification foci, or hemorrhage were detected inside the intra-axial well-defined lesion of the cysts, which appeared hypo-intense on T1 (Figure 1) and hyper-intense on T2 (Figure 2), with suppression of signal on FLAIR (Figure 3).

 

 

**Figure 4:**
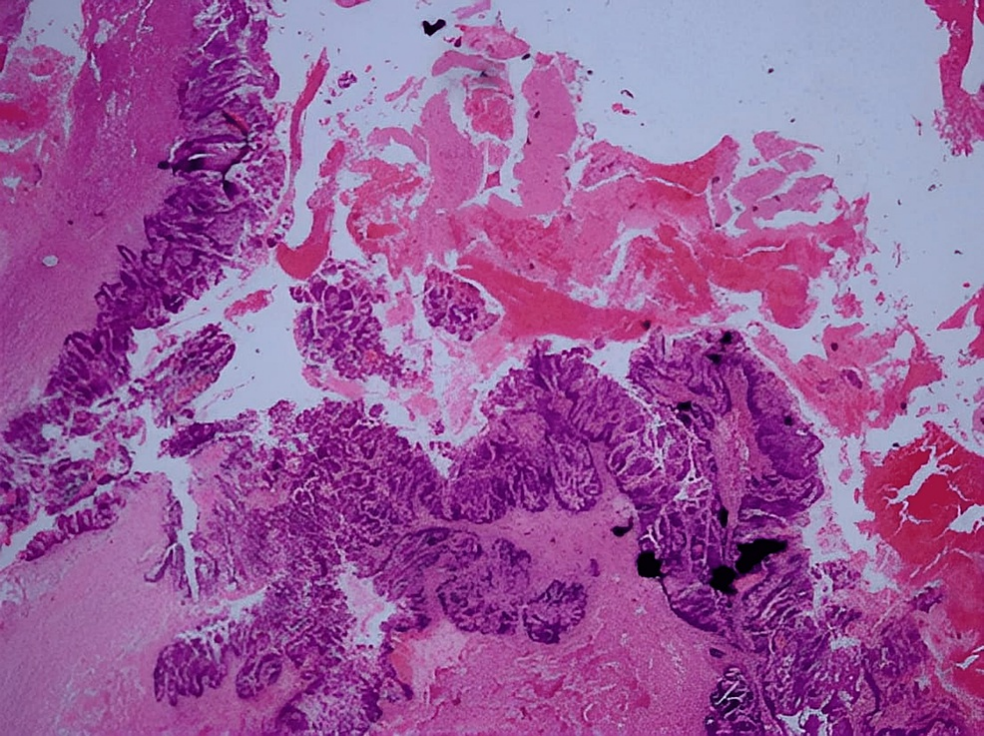
Cyst wall lined by flattened epithelium (H and E stain, 400x)

These imaging characteristics led to the conclusion that the lesion was a GEC. The cyst wall was lined with flattened cuboidal epithelium, according to the microscopic analysis of the H and E stained slide (Figure 4).

Neuroglial and adipose tissues may be seen in the subepithelium. The histological characteristics indicated that the disease was a GEC. With the progression of symptoms, the surgeon performed a transcortical craniotomy, and the cyst wall was fenestrated. A large portion of the wall was resected. Intraoperatively, a clean fluid oozed out when the cyst wall was opened. The patient tolerated the surgery well, and during the six-week postoperative appointment, there was a complete resolution of the pre-operative signs and symptoms. A microscopic examination of the cyst wall showed a ciliated cuboidal ependymal lined tissue resting over a thin layer of glial tissue.

## Discussion

GECs are uncommon, non-neoplastic, and epithelial-lined conditions in the central nervous system [2]. The most common site of these cysts is in the frontal lobe. However, they can be detected anywhere along the neural axis. They are congenital cystic lesions that develop due to fluid-filled embryonic neural tube components trapped in white matter [2]. Among all intracranial cysts, GECs make up less than 1% [3]. Children and newborns account for the majority of cases documented to date. The origin of this cyst and details about it need to be clarified [4-7]. These are often congenital lesions that develop into a fluid-filled hollow bordered with glial cells in the white matter from the sequestration of neural tube embryonic components. In our scenario, neuroglial heterotopia may have contributed to cysts. They could have developed due to the incorrect displacement of the normal ventricular epithelium at various stages of embryogenesis. It could be caused by prenatal injury and its aftereffects. The ependymal secretory activity determines how a cyst grows [8]. The clinical appearance was probably triggered by recent rapid growth because the cyst did not likely increase from embryogenesis until its beginning. The majority of GECs have no symptoms. Due to compression and mass influence on nearby neuroparenchyma, they could exhibit clinical symptoms. If the cysts are found inside the ventricular system, they might also impair the CSF circulation system and lead to hydrocephalus [3]. The patient’s clinical presentation will depend on the location of the cyst. Increased micturition is the presenting symptom if it is in the intramedullary cavity [4]. The presentation takes the form of compression over cranial nerves if it is close to the cerebellopontine angle [9]. CT scan and MRI imaging are the primary diagnostic modalities for these cysts. A well-defined T1 hypo-intense and T2 hyper-intense lesion with suppression may be detected on FLAIR images. A signal is homogeneous, occasionally somewhat higher than CSF, presumably because of the higher amount of protein in the cyst. Similar to other cases, ours had the characteristics of GECs. Complete cyst removal by the surgical method is the gold-standard treatment [10]. Another treatment option includes fenestration, but this results in the reoccurrence of the cyst. Cyst peritoneal shunt is a procedure similar to a ventriculoperitoneal shunt in which a cyst fluid is diverted to other sites to reduce intracranial pressure, but this procedure requires constant monitoring [11].

## Conclusions

GECs are rare but significant conditions that can cause various symptoms, including headaches, dizziness, and nausea. Early diagnosis and prompt treatment are essential for a positive outcome. In this case, surgical removal of the cyst was successful, and the patient experienced significant improvement in his symptoms. Regular follow-up appointments are critical to ensure that the patient continues to recover and that there are no signs of recurrence.
